# Central nervous system vasculitis mimicking a brain tumor: A case report

**DOI:** 10.1016/j.radcr.2025.11.068

**Published:** 2025-12-17

**Authors:** Uyen N.T. Nguyen, David Vo, Kari Hird, Victoria Wu, Vanessa Goodwill, Nikdokht Farid

**Affiliations:** a School of Medicine, University of California San Diego, La Jolla, CA 92093, USA; b Department of Neurosciences, University of California San Diego, La Jolla, CA 92093, USA; c Department of Pathology, University of California San Diego, La Jolla, CA 92093, USA; d Department of Radiology, University of California San Diego, La Jolla, CA 92093, USA

**Keywords:** CNS vasculitis, Primary angiitis of the central nervous system, Heterozygous RAG2 mutation

## Abstract

Central nervous system (CNS) vasculitis is a rare inflammatory condition of blood vessels of the brain and spinal cord that can result in profound neurological symptoms mimicking many other pathologies. We present the case of a 36-year-old male who presented with subacute onset of progressive left-sided hemiparesis and refractory seizures. The diagnosis was challenging and required comprehensive review and integration of different imaging modalities, tissue sampling, and biochemical testing. This case highlights key imaging features which may aid in distinguishing central nervous system vasculitis from other etiologies such as malignancy.

## Introduction

Primary CNS vasculitis, also called primary angiitis, is an inflammatory condition affecting mainly small- and medium-sized blood vessels of the brain or the spinal cord, without evidence of systemic inflammation. Its prevalence is rare with only 2.4 cases per million person-year [1]. This disease affects mostly middle-aged people with a median age of onset of 50 [[2], [3], [4]]. Primary CNS vasculitis is also a recognized but rare cause of ischemic stroke [5,6], with a reported prevalence of approximately 0.02% among patients admitted for stroke in a 10-year cohort study conducted in Australia [7]. Clinical presentation is usually nonspecific with symptoms such as headache, altered mental status, seizure, and stroke-like symptoms, which overlap with many other neurologic disorders [2]. Serum and CSF biomarkers have limited value for diagnosing CNS vasculitis due to low specificity and sensitivity, but thorough laboratory investigation is warranted to rule out other related etiologies such as malignancy, infection, systemic inflammation, or connective tissue disorders [8,9]. In addition to the clinical picture and laboratory analysis, the diagnosis of primary CNS vasculitis requires incorporation of imaging findings and tissue sampling. Different imaging modalities have been used, including CT, MRI, and digital subtraction angiography (DSA), with varying degrees of sensitivity and specificity [3]. According to the European Stroke Organization guidelines, DSA is recommended as the reference standard angiographic modality, particularly when CT/MR angiography are nondiagnostic, such as in cases of suspected medium-vessel involvement [10]. However, brain biopsy with histopathological classification remains the gold standard for diagnosing CNS vasculitis. The diagnostic criteria for CNS vasculitis include undefined acquired neurological impairments, evidence of inflammatory processes in arteries of the CNS, and ruling out secondary causes of systemic vasculitis [8]. CNS vasculitis is often a diagnosis of exclusion in clinical practice, and it remains challenging to suggest the diagnosis noninvasively. Early recognition is crucial to prompt clinical intervention, and thus, to improve patient outcomes.

We present a case of a young patient, who presented with subacute focal neurological deficits and new onset seizures. The initial imaging showed a brain lesion surrounded by vasogenic edema suspicious for possible malignancy. However, subsequent imaging suggested an inflammatory process rather than a brain tumor. The patient underwent comprehensive laboratory workup, biopsy, and eventually genetic testing which revealed a genetic condition that confirmed the diagnosis of primary CNS vasculitis. The novelty of this case lies in its distinctive imaging features, namely the delayed onset of restricted diffusion and rapid progression of vasogenic edema, as well as its association with a genetic condition not previously reported among tumor-mimic CNS vasculitis cases.

## Case report

A 36-year-old male with history of stroke, hypertension, type I diabetes, and end-stage renal disease (on hemodialysis) who initially presented with left sided weakness and focal seizures with secondary generalization. His initial head CT was reported as negative for mass lesion, hydrocephalus, or large transcortical infarct. The following day, he had another seizure and presented to a different hospital, where a brain MRI was obtained which revealed abnormal cortical swelling and FLAIR hyperintense signal in the right paramedian frontal lobe which was thought to be related to recent seizure activity. A few days later, he had a generalized seizure and repeat brain MRI showed persistent cortical swelling and signal abnormality in the right paramedian frontal lobe with new mild adjacent vasogenic edema which again was thought to be related to seizure activity. He was ultimately discharged on medical treatment for seizures.

Weeks later, he developed progressive weakness in the left hemibody. On exam, he had significant left face, arm and leg weakness with brisk reflexes in the left hemibody. Repeat MRI demonstrated new gyriform diffusion restriction in the right paramedian frontal lobe, corresponding to the previously noted region of cortical swelling and signal abnormality, as well as significantly increased vasogenic edema surrounding this region (Fig. 3), which was also evident on the repeated head CT (Fig. 2). Additionally, MR spectroscopy was performed and demonstrated doublet lactate peak and mildly increased Cho/NAA ratio (Fig. 3).

Concurrently, extensive serum studies were initiated, with unremarkable results for infection (negative for syphilis, tuberculosis, and HIV), sarcoidosis (unremarkable serum angiotensin-converting enzyme), antiphospholipid syndrome (unremarkable serum antinuclear antibody, antineutrophil cytoplasmic antibodies, cardiolipin and beta 2 glycoprotein IgM/IgG, dilute Russell’s viper venom time), and systemic vasculitis syndromes (negative for cryoglobulin and ANCA).

Cerebral spinal fluid (CSF) analysis was pertinent for red blood cells (151mm^3^, lab ref. 0mm^3^) and glucose (101 mg/dL, lab ref. 40-70 mg/dL), but revealed unremarkable nucleated cells (<1 mm^3^, lab ref. 0-5 mm^3^), unremarkable protein (36, lab ref. 15-45 mg/dL), normal lactate (1.9, lab ref. 1.1-2.4 mmol/L), normal pyruvate (0.134, lab ref. 0.060-0.190 mmol/L), normal cytokine panel, and negative oligoclonal bands. Infectious studies were also unremarkable, including negative CSF gram stain/culture, meningitis/encephalitis panel, coccidioides, syphilis, West Nile virus, cryptococcus, and HSV. CSF hematopathology was also negative. CT chest, abdomen and pelvis did not reveal any evidence of primary malignancy.

Notably, the patient was found to have deficiencies in immunoglobulins A, M, and G, and was heterozygous for a RAG2 mutation, which is associated with autosomal recessive severe combined immunodeficiency.

The patient ultimately underwent a brain biopsy with pathology revealing occlusive vasculopathy and microangiopathy with evidence of acute/subacute ischemic necrosis of brain tissue. Subsequently, a diagnostic cerebral angiogram (DSA) was performed, which revealed subtle irregularities of the distal branches of the anterior circulation without medium- or large-vessel involvement (Fig. 4).

Therefore, the patient was diagnosed with primary CNS vasculitis. He was treated with 5 days of pulse dose Dexamethasone 4mg IV every 12 hours, which led to significant improvement in strength of his left hemibody. He was discharged on Prednisone taper (60 mg daily for 30 days, then tapered by 10 mg every 7 days to 10 mg) and demonstrated continued clinical improvement in both mobility and strength. The patient remained seizure-free at the 1-month outpatient follow-up.

## Discussion

**Fig. 1:**
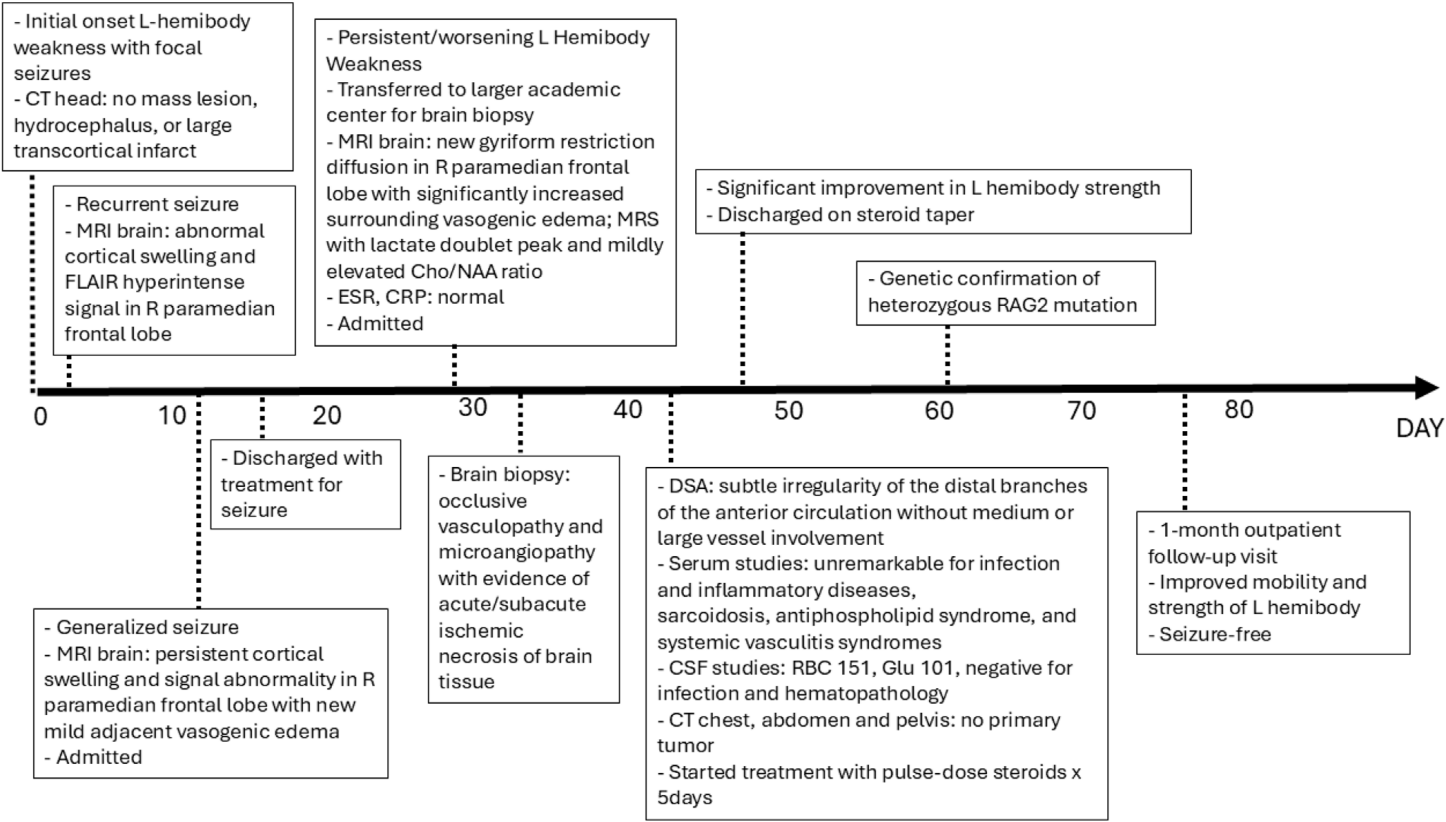
Clinical timeline overview. Abbreviations: Cho/NAA ratio, Choline (Cho) to N-acetylaspartate (NAA) ratio; CRP, C-reactive protein; CSF, cerebrospinal fluid; CT, computed tomography; DSA, digital subtraction angiography; ESR, Erythrocyte Sedimentation Rate; FLAIR, fluid-attenuated inversion recovery; Glu, Glucose; L, left; MRI, magnetic resonance imaging; MRS, magnetic resonance spectroscopy; R, right; RBC, red blood cell.

We present a unique and challenging case of primary CNS vasculitis. This condition may present with clinical and imaging features that mimic other diseases such as acute stroke or brain malignancy. Our patient presented with refractory seizures and progressive left-sided body weakness (Fig. 1). His symptoms and past medical history, which included vascular risk factors, were highly concerning for an acute stroke. However, sequential MRIs of the brain demonstrated worsening changes in the right paramedian frontal lobe starting with cortical swelling and signal abnormality and progressing to gyriform restricted diffusion and significant surrounding vasogenic edema. Although acute stroke and brain tumor were initially high on the differential, there are 2 imaging features in our case that support the ultimate diagnosis of CNS vasculitis: the delayed restricted diffusion and the progressive vasogenic edema surrounding this region.

**Fig. 2:**
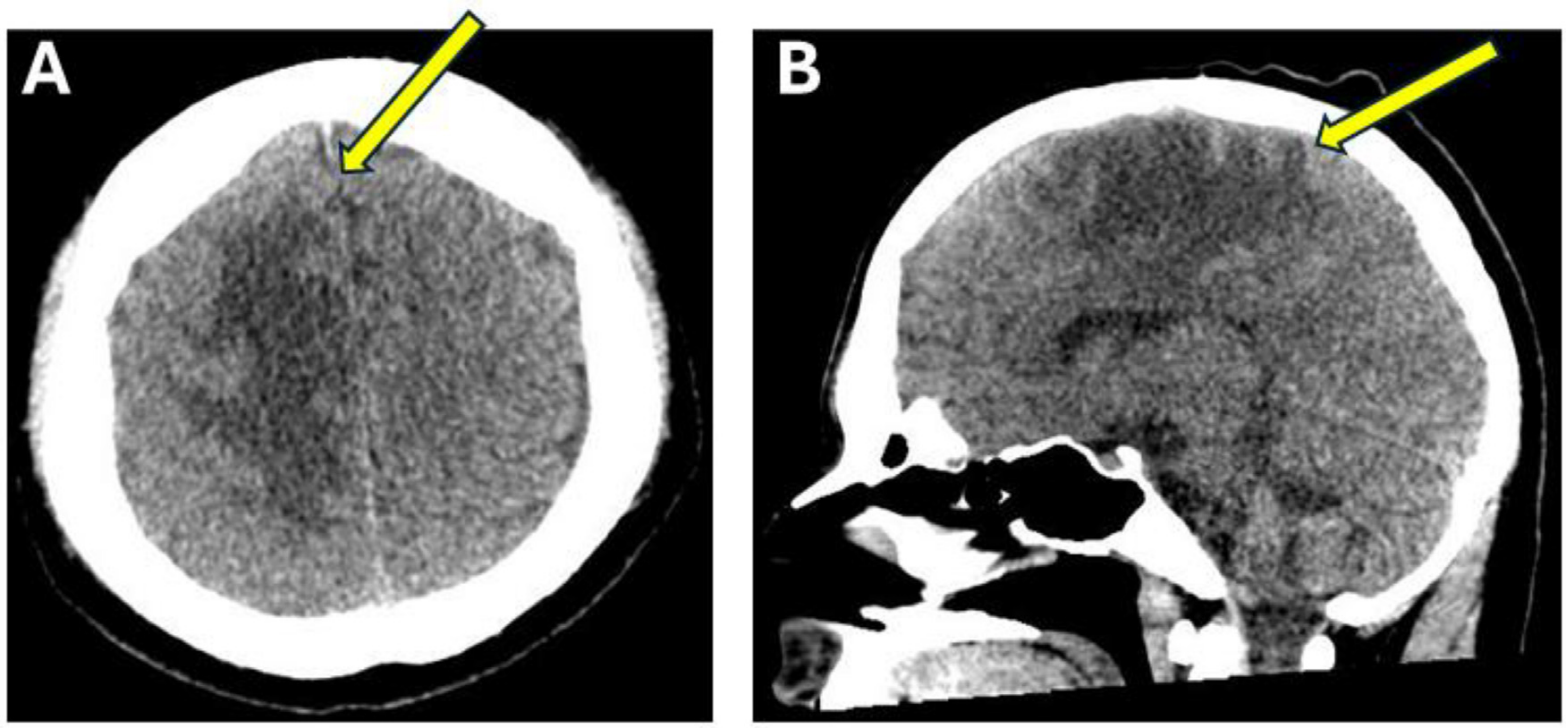
Head CT preceding the MRI obtained after the patient was transferred to the large academic center. Axial (A) and sagittal (B) head CT images demonstrate a large area of vasogenic edema (arrows) throughout the paramedian right frontal and parietal lobe.

**Fig. 3:**
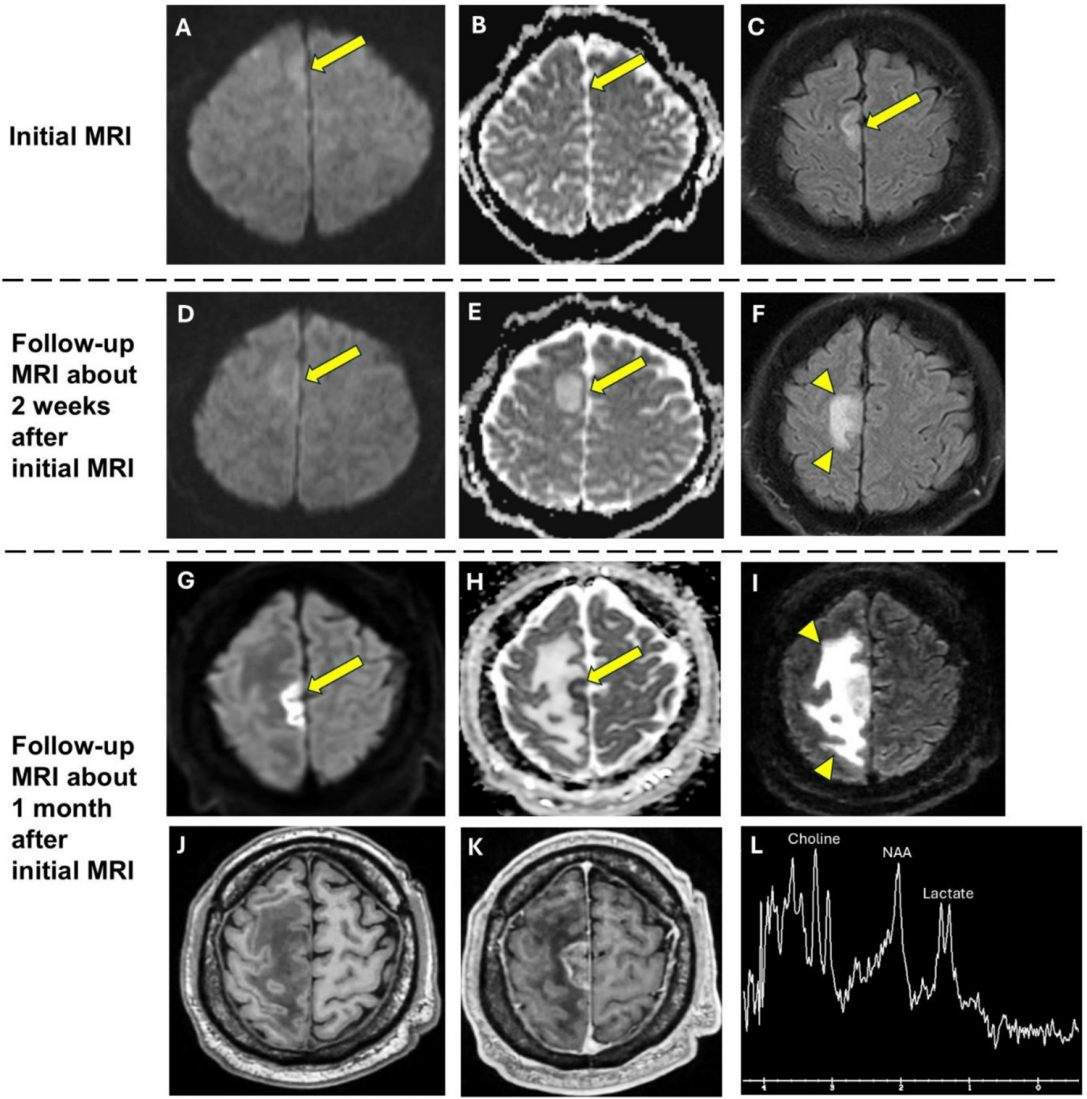
Three sequential brain MRI studies showing temporal progression of vasogenic edema and the delayed restricted diffusion in right paramedian frontal lobe. Initial MRI: Diffusion weighted image (A) and Apparent diffusion coefficient image (B) demonstrate focal abnormal cortical swelling (arrows) and FLAIR image (C) demonstrates hyperintense signal (arrow) in right paramedian frontal lobe. No associated restricted diffusion. Follow-up MRI about 2 weeks after initial MRI: Diffusion weighted image (D) and Apparent diffusion coefficient image (E) demonstrate persistent cortical swelling and signal abnormality (arrows) in right paramedian frontal lobe with new mild adjacent vasogenic edema (arrowheads) on FLAIR image (F). No associated restricted diffusion. Hyperintense signal on Apparent Diffusion Coefficient image (E) is compatible with vasogenic edema. Follow-up MRI about 1 month after initial MRI: Diffusion weighted image (G) and apparent diffusion coefficient image (H) demonstrate a region of restricted diffusion (arrows) in the posterior paramedian right frontal lobe. FLAIR image (I) demonstrates a large area of vasogenic edema (arrowheads) surrounding the area of restricted diffusion. T1-weighted precontrast (J) and T1-weighted postcontrast (K) images demonstrate heterogeneous enhancement within the area of restricted diffusion. MR Spectroscopy (L) demonstrates doublet lactate peak and mildly elevated Choline/NAA ratio.

Although restricted diffusion on brain MRI is most commonly the hallmark of acute infarct, this feature can be observed in other pathologic entities including brain tumors such as lymphoma [9] and glioblastoma [11]. In our case, the gyriform restricted diffusion was not present on the initial MRI studies but became apparent on subsequent imaging (Fig. 3). While restricted diffusion has been described in cases of CNS vasculitis [[12], [13], [14]], the occurrence of delayed restricted diffusion has not been previously reported and may represent a unique finding in our case. This may be due to the rapid progression of inflammation in CNS vasculitis which can lead to progressive narrowing and blockage of smaller vessels, ultimately resulting in tissue hypoxia as the inflammation becomes more pronounced. In contrast, restricted diffusion in a brain tumor results from increased cellularity of the tumor, particularly with highly cellular tumors such as lymphoma.

The progressive vasogenic edema surrounding the area of restricted diffusion was also more suggestive of an inflammatory process. While increased permeability from damaged blood-brain barrier in the setting of inflammation causes vasogenic edema, an acute infarct is characterized by cytotoxic edema [15]. Although vasogenic edema is also commonly associated with brain tumors, the rapid progression of edema within a short amount of time (ie 2 weeks) seen in our case would be atypical for a brain tumor. This feature is more suggestive of a rapidly progressive inflammatory process such as CNS vasculitis.

MR spectroscopy may play a crucial role in differentiating malignant from nontumor lesions. While the choline peak reflects cell membrane turnover, the NAA peak reflects normal neural tissue. The Choline/NAA ratio is usually greater than 1.9 in brain tumors [16]. Other processes associated with increased cellular turnover, such as demyelinating diseases [17] and CNS vasculitis [18], can also elevate the Choline/NAA ratio, however to a lesser degree. In our case, there was only a mildly increased Choline/NAA ratio (Fig. 3), less than 1.5, making brain tumor less likely.

**Fig. 4:**
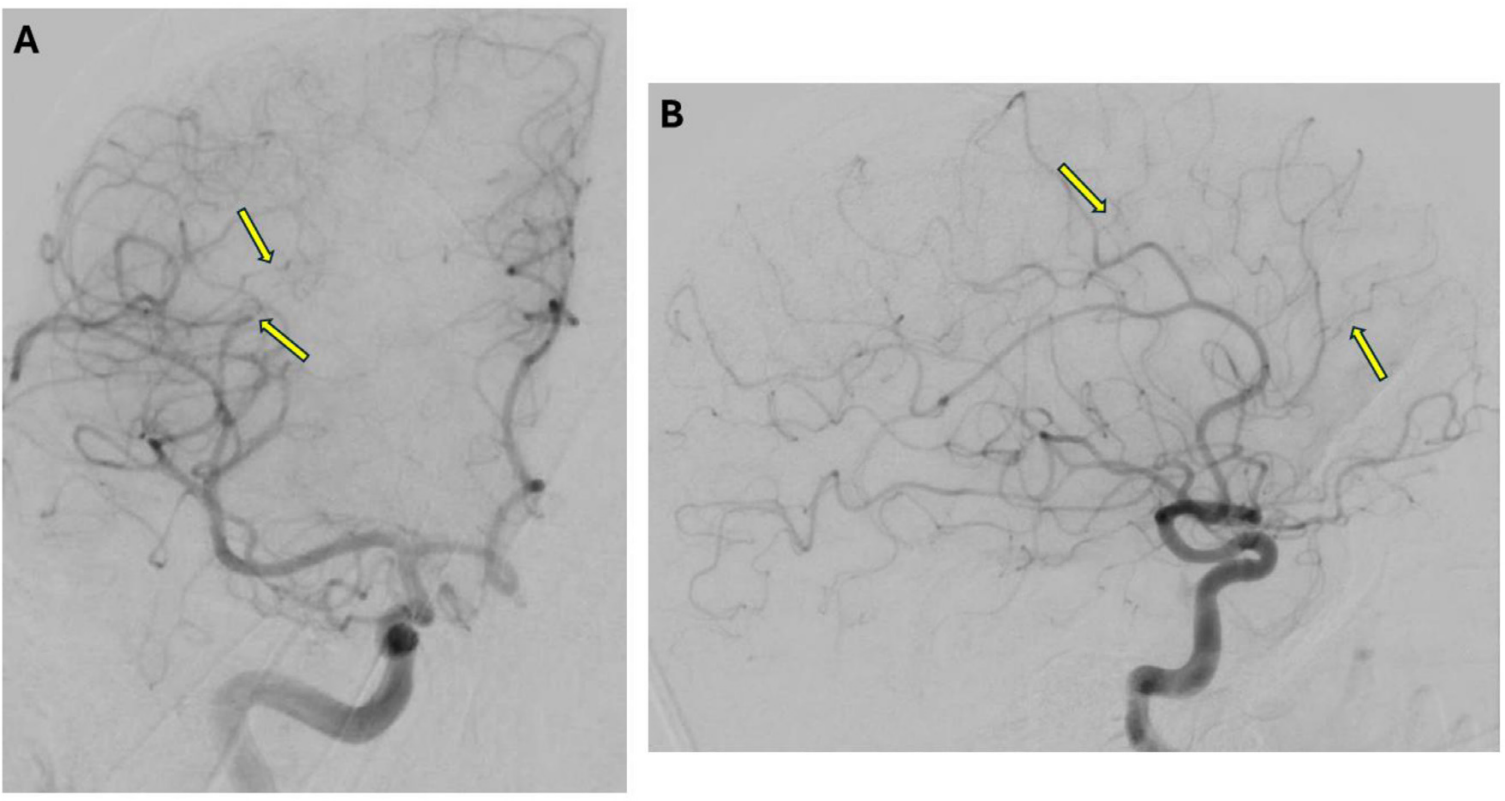
Digital subtraction angiography (5 vessel angiogram of right ICA, right ECA, left ICA, left ECA and left vertebral artery with contrast use of 60 mL of Omnipaque 240). Frontal (A) and Lateral (B) projections following contrast injection into the right ICA showing subtle irregularities (arrows) of the distal branches of the anterior circulation without medium or large vessel involvement.

In general, MRI is sensitive in cases of CNS vasculitis [7,8,19], however the imaging findings are nonspecific and include white matter abnormalities [3,20], infarction [3,21,22], and petechial hemorrhage [22,23]. CNS vasculitis may also mimic a brain tumor, so-called pseudotumoral CNS vasculitis, which is seen in about 15% of cases [3,24,25]. MR vessel wall imaging is another imaging modality that may be utilized to evaluate for CNS vasculitis [26]. Although vessel wall imaging was performed in our case, the study was negative without abnormal vessel wall enhancement. In addition to MRI, CT/MR angiography may be obtained, but is less sensitive for detecting small-vessel vasculitis [3]. On the other hand, DSA is highly sensitive in detecting vasculitis, especially for small-vessel and medium-vessel vasculitis, which makes it the “gold standard” imaging modality for CNS vasculitis [4,22]. In our case, DSA demonstrated subtle irregularities of the distal small blood vessels (Fig. 4), which helped to confirm the diagnosis.

**Fig. 5:**
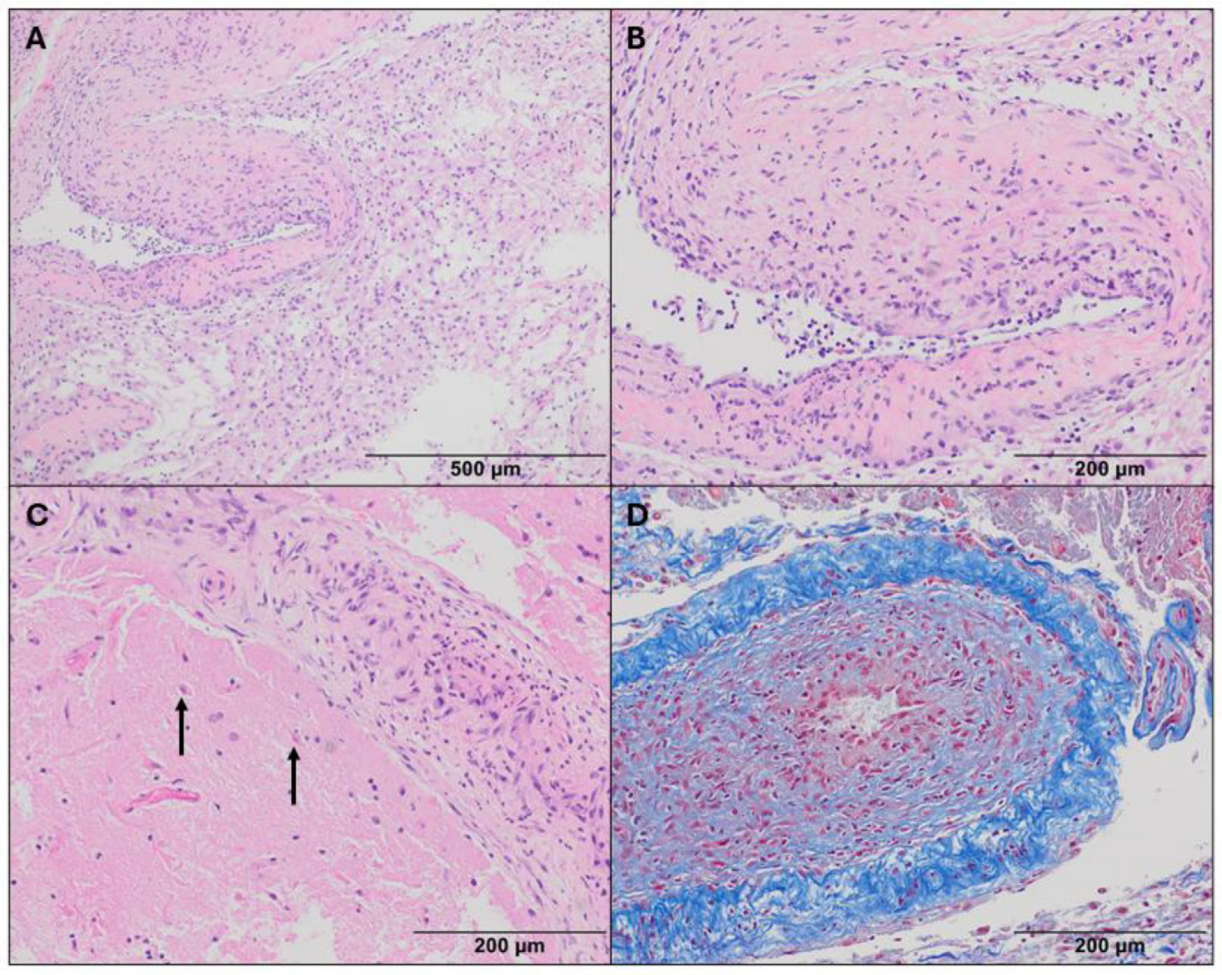
Biopsy pathology. An abnormal vessel with prominent luminal thickening is surrounded by a sheet of macrophages indicating subacute necrosis (A, H&E 100x). At higher magnification, neutrophils are seen within the thickened vessel wall (B, H&E 200x). Some vessels were completely occluded and surrounded by acutely ischemic hypereosinophilic “red dead” neurons (arrow) (C, H&E 200x). A trichrome stain highlights fibrous intimal vascular thickening (D, 200x).

Brain biopsy is usually required for confirming CNS vasculitis as well as excluding other diseases such as malignancy, as in our case. Histopathological presentation of CNS vasculitis is varied but can be classified into 3 main subtypes: granulomatous, necrotizing, and lymphocytic [8]. The pathological findings in our case included cavitating subacute necrosis, infiltration of macrophages and neutrophils, and rare lymphocytes (Fig. 5), suggestive of an atypical necrotizing subtype. On a macroscopic level, there was narrowing of leptomeningeal medium-sized vessels and parenchymal small-sized vessels.

Another unique feature of this case is that the patient was later confirmed to have a genetic condition, which may be a predisposing factor for developing CNS vasculitis at a younger age. Our patient was found to be heterozygous for the RAG2 gene. This gene, together with the RAG1 gene, encodes for Recombination Activating Gene proteins, which play a critical role in immunity. They allow for recombination of genes at the receptors of T-cells to generate both T-cells and B-cells [27]. Heterozygosity of RAG2 mutation results in partial RAG deficiency causing immune dysregulation [28]. Patients with partial RAG deficiency can present with a variety of autoimmune-related conditions. Among these, vasculitis has been recognized as the fourth most common with relatively high morbidity [27,29]. In the literature, only 1 case of CNS vasculitis with partial RAG2 deficiency has been described [28] but did not present with imaging findings mimicking tumor as in our case.

Although there is no current established standardized therapeutic regime for primary CNS vasculitis, immunosuppressive monotherapy with glucocorticoids or in combination with cyclophosphamide or rituximab can provide rapid symptom improvement and is effective in inducing long-term remission [4,30]. Before initiation of immunosuppression therapy for suspected CNS vasculitis, it is critical to thoroughly exclude other disease entities [4]. Our patient was treated with steroids after histopathological confirmation of CNS vasculitis with significant clinical improvement in a short amount of time.

In summary, we present a unique case of CNS vasculitis with rapid progression both clinically and on imaging. Although the imaging findings of CNS vasculitis are nonspecific, it is important to consider this diagnosis particularly when imaging findings, such as delayed restricted diffusion and worsening vasogenic edema, suggest an evolving inflammatory process. Although DSA can be very helpful in making the diagnosis, often brain biopsy is required for confirmation, as in our case. Additionally, our case highlights the association of CNS vasculitis with genetic abnormalities in the RAG genes and the importance of checking for this genetic abnormality in suspected cases of CNS vasculitis.

## Patient consent

Written informed consent was obtained from the patient for relevant clinical data and images regarding the medical condition described in this case report to be used and published in this journal.

## Author contributions

All authors contributed to the acquisition, analysis, and interpretation of data; participated in the drafting and revision of the manuscript; and approved the final version for publication.

## References

[1] Salvarani C, Brown RS, Calamia KT, TJH Christianson, Weigand SD, Miller DV, et al. (2007). *Primary central nervous system vasculitis: analysis of 101 patients*. 62(5), 442–451. 10.1002/ana.21226 17924545

[2] Salvarani C., Brown R.D., Hunder G.G. (2012). Adult primary central nervous system vasculitis. The Lancet.

[3] Abdel Razek A.A.K., Alvarez H., Bagg S., Refaat S., Castillo M. (2014). Imaging spectrum of CNS vasculitis. RadioGraphics.

[4] Kraemer M., Berlit P. (2021). Primary central nervous system vasculitis: an update on diagnosis, differential diagnosis and treatment. J Neurolog Sci.

[5] Kleindorfer D.O., Towfighi A., Chaturvedi S., Cockroft K.M., Gutierrez J., Lombardi-Hill D. (2021). 2021 guideline for the prevention of stroke in patients with stroke and transient ischemic attack: a guideline from the American Heart Association/American Stroke Association. Stroke.

[6] Campos A.C., Sarmento S., Narciso M., Fonseca T. (2023). Primary central nervous system vasculitis: a rare cause of stroke. Cureus.

[7] Kempster P.A., McLean C.A., Phan T.G. (2016). Ten year clinical experience with stroke and cerebral vasculitis. J Clin Neurosci.

[8] Godasi R., Bollu P.C. (2020). https://www.ncbi.nlm.nih.gov/books/NBK482476/.

[9] Zacharia T.T., Law M., Naidich T.P., Leeds N.E. (2008). Central nervous system lymphoma characterization by diffusion-weighted imaging and MR spectroscopy. J Neuroimag.

[10] Pascarella R., Antonenko K., Boulouis G., De Boysson H., Giannini C., Heldner M.R. (2023). European Stroke Organisation (ESO) guidelines on primary angiitis of the central nervous system (PACNS). Eur Stroke J.

[11] Gupta A., Young R.J., Karimi S., Sood S., Zhang Z., Mo Q. (2011). Isolated diffusion restriction precedes the development of enhancing tumor in a subset of patients with glioblastoma. Am J Neuroradiol.

[12] White M.L., Hadley W.L., Zhang Y., Dogar M.A. (2007). Analysis of central nervous system vasculitis with diffusion-weighted imaging and apparent diffusion coefficient mapping of the normal-appearing brain. Am J Neuroradiol.

[13] Salvarani C., Brown R.D., Christianson T., Miller D.V., Giannini C., Huston J. (2015). An update of the Mayo Clinic cohort of patients with adult primary central nervous system vasculitis: description of 163 patients. Medicine.

[14] Sheikh T.S., Rozenberg A., Merhav G., Shifrin A., Stein P., Shelly S. (2024). Primary CNS vasculitis: insights into clinical, neuropathological, and neuroradiological characteristics. Front Neurol.

[15] Michinaga S., Koyama Y. (2015). Pathogenesis of brain edema and investigation into anti-edema drugs. Int J Mol Sci.

[16] Majós C., Aguilera C., Alonso J., Julià-Sapé M., Castañer S., Sánchez J.J. (2009). Proton MR spectroscopy improves discrimination between tumor and pseudotumoral lesion in solid brain masses. Am J Neuroradiol.

[17] Ikeguchi R., Shimizu Y., Abe K., Shimizu S., Maruyama T., Nitta M. (2018). Proton magnetic resonance spectroscopy differentiates tumefactive demyelinating lesions from gliomas. Multiple Scleros Relat Disord.

[18] Lu P., Cui L., Zhang L., Wang H., Yin L., Tian D. (2025). Magnetic resonance spectroscopy for discriminating primary angiitis of the central nervous system from gliomas and lymphomas. Acta Neurologica Belgica.

[19] Agarwal A., Sharma J., Srivastava M.V.P., Sharma M.C., Bhatia R., Dash D. (2022). Primary CNS vasculitis (PCNSV): a cohort study. Scient Rep.

[20] Qin L., He M., Lu W. (2023). Case report: a case of primary angiitis of the central nervous system: misdiagnosed for 3.5 years. Front Neurol.

[21] Madhumati N., Chetan H. (2021). A rare case report of primary central nervous system vasculitis. APIK J Intern Med.

[22] Junek M., Perera K.S., Kiczek M., Hajj-Ali R.A. (2023). Current and future advances in practice: a practical approach to the diagnosis and management of primary central nervous system vasculitis. Rheumatol Adv Pract.

[23] Roberts J.I., Ng D., Kapadia R. (2024). Pearls & oy-sters: tumour-like mass lesion secondary to primary CNS vasculitis. Neurology.

[24] Qu S.B., Khan S., Liu H. (2009). Primary central nervous system vasculitis mimicking brain tumour: case report and literature review. Rheumatol Int.

[25] Suthiphosuwan S., Bharatha A., Hsu C.C., Lin A.W., Maloney J.A., Munoz D.G. (2020). Tumefactive primary Central nervous system vasculitis: imaging findings of a rare and underrecognized neuroinflammatory disease. Am J Neuroradiol.

[26] Salvarani C., Hunder G.G., Brown R.D. (2024). Primary Central nervous system vasculitis. New Engl J Med.

[27] Delmonte O.M., Schuetz C., Notarangelo L.D. (2018). RAG deficiency: two genes, many diseases. J Clin Immunol.

[28] Delmonte O.M., Villa A., Notarangelo L.D. (2020). Immune dysregulation in patients with RAG deficiency and other forms of combined immune deficiency. Blood.

[29] Geier C.B., Farmer J.R., Foldvari Z., Ujhazi B., Steininger J., Sleasman J.W. (2020). Vasculitis as a major morbidity factor in patients with partial RAG deficiency. Front Immunol.

[30] Byram K., Hajj-Ali R.A., Calabrese L. (2018). CNS vasculitis: an approach to differential diagnosis and management. Curr Rheumatol Rep.

